# Dissecting whole-brain conduction delays through MRI microstructural measures

**DOI:** 10.1007/s00429-021-02358-w

**Published:** 2021-08-14

**Authors:** Matteo Mancini, Qiyuan Tian, Qiuyun Fan, Mara Cercignani, Susie Y. Huang

**Affiliations:** 1 Department of Neuroscience, Brighton and Sussex Medical School, University of Sussex, Brighton, UK; 2 Cardiff University Brain Research Imaging Centre (CUBRIC), Cardiff University, Cardiff, UK; 3 NeuroPoly Lab, Polytechnique Montréal, Montréal, Canada; 4 Athinoula A. Martinos Center for Biomedical Imaging, Department of Radiology, Massachusetts General Hospital, Charlestown, MA USA; 5 Harvard Medical School, Boston, MA USA; 6 Harvard-MIT Division of Health Sciences and Technology, Massachusetts Institute of Technology, Cambridge, MA USA

**Keywords:** Brain networks, MRI, White matter, Conduction delays, Microstructure

## Abstract

Network models based on structural connectivity have been increasingly used as the blueprint for large-scale simulations of the human brain. As the nodes of this network are distributed through the cortex and interconnected by white matter pathways with different characteristics, modeling the associated conduction delays becomes important. The goal of this study is to estimate and characterize these delays directly from the brain structure. To achieve this, we leveraged microstructural measures from a combination of advanced magnetic resonance imaging acquisitions and computed the main determinants of conduction velocity, namely axonal diameter and myelin content. Using the model proposed by Rushton, we used these measures to calculate the conduction velocity and estimated the associated delays using tractography. We observed that both the axonal diameter and conduction velocity distributions presented a rather constant trend across different connection lengths, with resulting delays that scale linearly with the connection length. Relying on insights from graph theory and Kuramoto simulations, our results support the approximation of constant conduction velocity but also show path- and region-specific differences.

**Supplementary Information:**

The online version contains supplementary material available at 10.1007/s00429-021-02358-w.

## Introduction

Network models offer a powerful framework to study structure–function relationships in the human brain. Previous studies have highlighted similarities between structural and functional connectivity patterns (Honey et al., 2010; van den Heuvel et al., 2009), and simulation-based work has suggested the idea that structure can support and shape multiple functional configurations (Cabral et al., 2017). From the point of view of combining local dynamics with global interactions, these simulations can be logically separated into two components (Petkoski & Jirsa, 2019; Sanz-Leon et al., 2015): first, an oscillator used to model the behavior of neuronal populations in the gray matter; second, a set of rules describing how these oscillators interact with each other. The latter is where the structural connectivity estimated from white matter pathways comes into play. A fundamental and simple approach is given by the Kuramoto model (Kuramoto, 1984), that assumes the activity of a local system can be represented by their phase alone (Breakspear et al., 2010). In applications to brain dynamics, the Kuramoto model has been used both for slow oscillations as in metabolic activity (as estimated by functional magnetic resonance imaging), and fast ones as in electrical activity (as measured for example through magnetoencephalography) (Breakspear, 2017).

As the neuronal populations are distributed in space (i.e., through the cortical surface) and are interconnected by bundles of different length and with different features (e.g., myelination), assumptions about conduction velocity and therefore delays along those connections are needed to study global brain dynamics. As several studies focused on slow oscillations, a common assumption is that such conduction delays are negligible (de Lange et al., 2019; Gollo et al., 2017; Schmidt et al., 2015). In studies focused on faster oscillations, the conduction velocity is usually assumed to be constant, and therefore the delays scale linearly with the connection lengths (Cabral et al., 2011,2014). An alternative approach assumes instead that the delays are constant (Roberts et al., 2019), with higher conduction velocity compensating for longer connections. The validation of these assumptions is nontrivial: directly measuring delays not only involves invasive recordings with direct electrical stimulation (Silverstein et al., 2020), but also an extensive sampling effort over the whole cortex. An additional factor to consider is where the recordings and the stimulation are performed, as interfacing with the cortical surface leads to longer delays compared to directly accessing the white matter pathways (Shimono & Hatano, 2018).

Microstructural models fitted to magnetic resonance imaging (MRI) data provide a non-invasive alternative to directly measuring conduction delays. Such models exploit the sensitivity of MRI to biophysical quantities to estimate microscopic features of the neural tissue, and some of these features are determinants of conduction velocity. The first and fundamental determinant is the axonal diameter, as in myelinated axons conduction velocity is proportional to the inner (i.e., without accounting for the myelin sheath) diameter. Using diffusion MRI and ultra-high gradients (Huang et al., 2020; Jones et al., 2018), it is possible to estimate the axonal diameter in the human brain (at least for the largest ones), and recent studies have shown measures in line with the underlying histology (Huang et al., 2020; Veraart et al., 2020) and with good scan-rescan reliability (Veraart et al., 2021). Another important determinant of conduction velocity is the myelin content. The presence of the myelin sheath itself not only changes conduction dramatically, but also its amount increases velocity non-linearly (Drakesmith et al., 2019; Rushton, 1951). The specific contribution of myelin depends on the so-called g-ratio, which is the ratio between the inner and the outer diameter of the myelinated axon. While myelin indirectly influences diffusion phenomena, diffusion MRI is not specific to myelin content (Beaulieu, 2002), and therefore other techniques are required for quantitative measures. Fortunately, myelin influences several MRI-based measures (Piredda et al., 2021), including longitudinal and transverse relaxation rates, as well as magnetization transfer. These measures have generally been shown to be sensitive to myelin content according to the histological ground truth (Mancini et al., 2020). Using axonal diameter and myelin content measures, it is then possible to estimate the conduction velocity through single-neuron models (Arancibia-Cárcamo et al., 2017; Richardson et al., 2000) or through more simplified ones, such as the Rushton model (Rushton, 1951) or the Waxman model (Waxman & Bennett, 1972).

Leveraging these advanced MRI measures, in this study we aim to define whole-brain structural connectivity in terms of conduction delays, as estimated from microstructural measures. Our goal is first to characterize the conduction velocity and delay distributions as a function of the related connection lengths, and then to assess conduction delay approximations currently used in the literature.

## Results

We estimated a rich set of structural connectivity patterns in a group of healthy volunteers combining tractography and several microstructural measures: the axonal diameter, estimated from ultra-high gradient diffusion data using a spherical mean model (Fan et al., 2020); the myelin volume fraction, estimated as the macromolecular tissue volume (MTV) (S. Berman et al., 2018; Mezer et al., 2013); the g-ratio, computed using the approach proposed by Stikov and colleagues (Stikov et al., 2015); and finally, the conduction velocity, computed from the axonal diameter and the g-ratio (a relative measure of myelination) using the Rushton model (Rushton, 1951). All these measures were mapped onto the corresponding tractograms and subsequently used to weight the respective connectivity matrices, based on regions of interest (ROIs) from the Desikan-Killiany (Desikan et al., 2006) and the Lausanne (Cammoun et al., 2012) atlases, which were averaged to obtain group-level connectivity. Conduction delays were computed at the subject level as the ratio between the average connection length and the correspondent average conduction velocity for each connection.

**Fig. 1:**
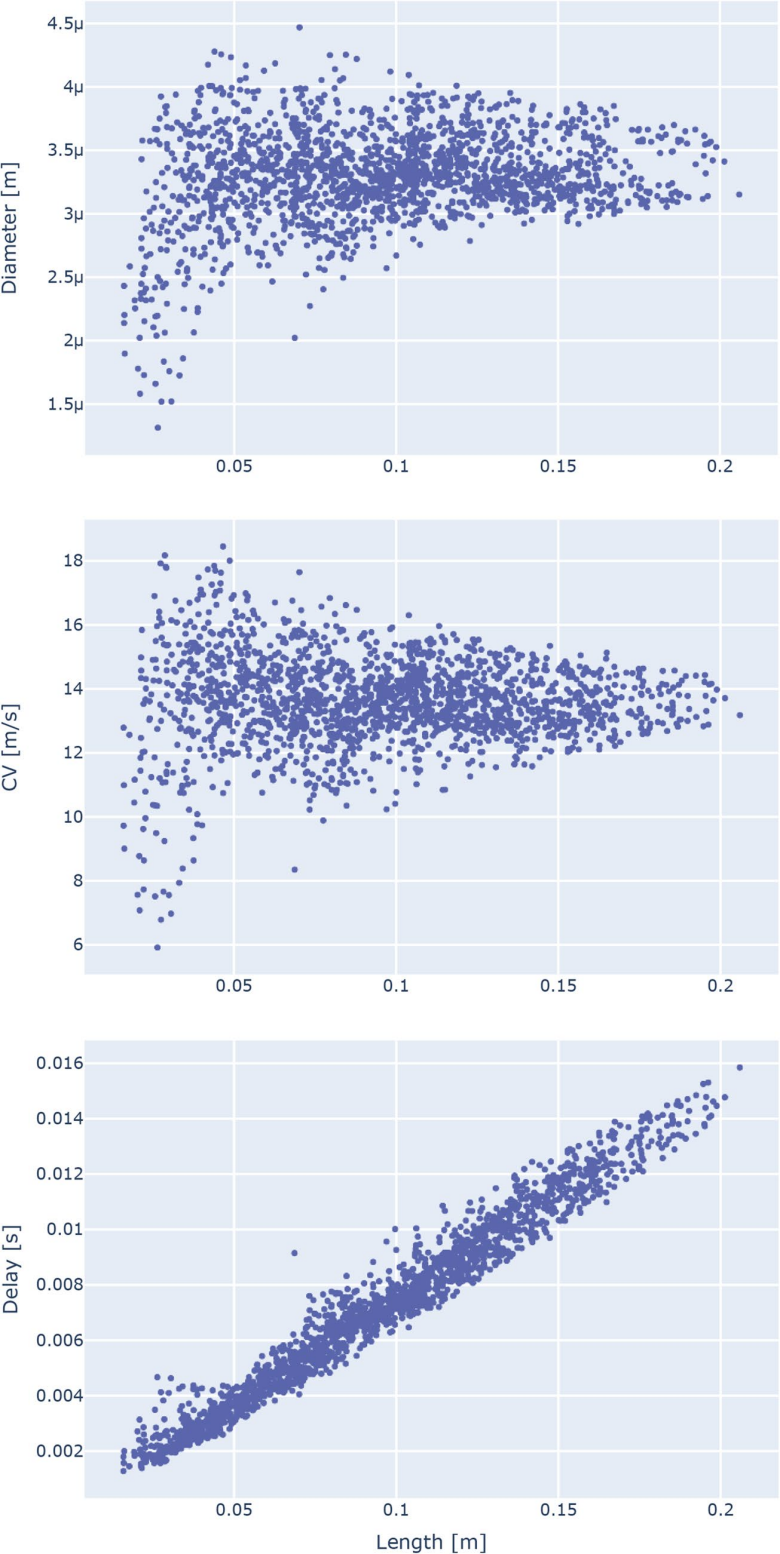
Distributions of the axonal diameter (top), conduction velocity (middle) and delay (bottom) as a function of the connection length. Although the conduction velocity takes into account both the diameter and the myelin content, the plot strongly resembles the diameter one. Since the delay is the ratio between the length and the conduction velocity, the overall constant velocity trend results in a linear relationship. Each point in the scatterplots represents a connection in the group network

**Fig. 2:**
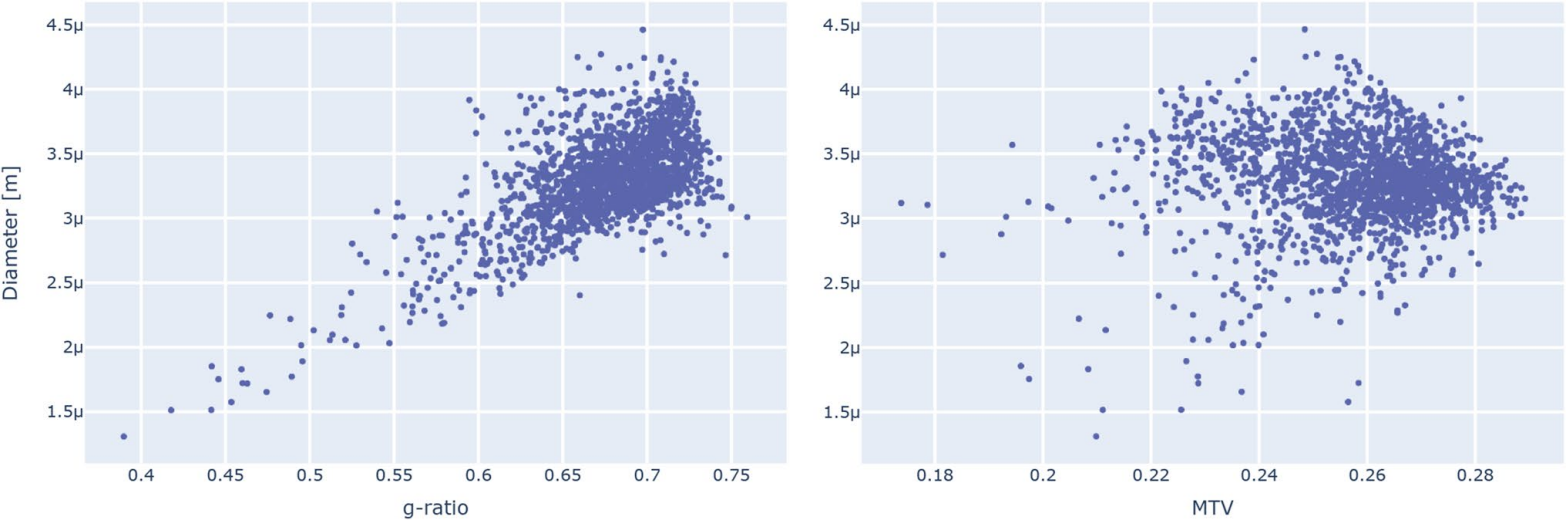
Axonal diameter distributions as a function of the g-ratio (left) and MTV (right). The g-ratio and the diameter have a pronounced linear relationship, while MTV (which is an absolute measure of myelin) does not present a clear trend

As the first fundamental step, we characterized the relationships between each microstructural measure along a connection and its respective length (Fig. 1). Looking at the axonal diameter distribution as a function of connection length, we observed that most connections (83.95%) have an average diameter between 3 μm and 4 μm, with only very short connections (shorter than 0.05 m) presenting a more pronounced variability. Regarding the relationship between axonal diameter and myelin content (Fig. 2), we observed a linear trend between the g-ratio and the diameter (linear regression R^2^: 0.4263). Interestingly, when looking at the myelin volume fraction, which is an absolute measure of myelin, the trend disappears.

We then characterized the conduction velocity estimated using the Rushton formula in terms of connection length (Fig. 1—middle). The trend is very similar to the one between axonal diameter and length (Pearson’s correlation coefficient: 0.8317; the relationships between conduction velocity and, respectively, axonal diameter and g-ratio are showed in Figure S1), where for most connections the velocity distribution follows a constant trend. As a result, the conduction delay shows a highly linear relationship with the connection length (linear regression R^2^: 0.9635), with an angular coefficient equal to 0.07447. These trends were conserved when using a denser parcellation scheme such as the Lausanne atlas (Figure S2), and also at the single-subject level (Figure S3-S7).

**Fig. 3:**
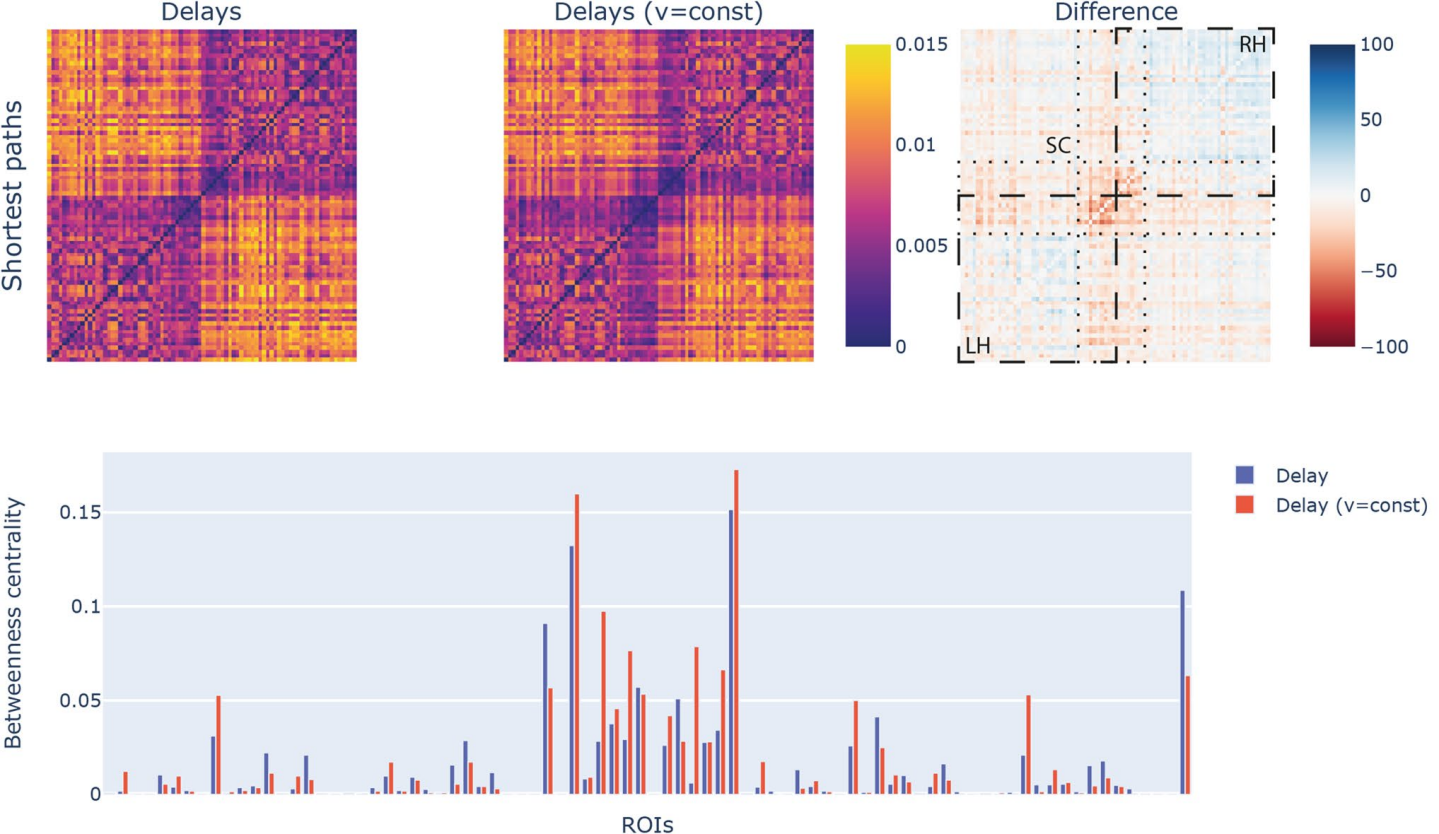
Comparisons between conduction delay distribution with the constant velocity approximation using graph measures: the shortest paths for the delay distribution (top-left) are distributed similarly to the constant velocity case (top-middle), but in terms of quantitative differences there are pronounced mismatches for subcortical connections (top-right); similarly, the betweenness centrality of each node (bottom) shows cases of both underestimation and overestimation. The ROIs are detailed in the supplementary material (Table S1). On the difference matrix we highlighted the left (LH) and right (RH) intra-hemispheric connections (dashed lines) as well as the subcortical (SC) connections (dotted lines)

The reciprocal of the angular coefficient from the length-delay relationship provides an estimated conduction velocity of 13.42 m/s. Using this value, we computed a delay distribution on the basis of the observed connection lengths under the assumption of fixed conduction velocity. In this way, we were able to compare the differences with the actual delay distribution computed from the axonal diameter values. For this comparison, we started analyzing path measures borrowed from the field of graph theory (Fig. 3). We first computed the shortest paths of the network between each pair of nodes for the two delay distributions. Despite being qualitatively similar, the delay distribution based on constant velocity shows shorter paths (25–50%) for subcortical connections. Taking into account the betweenness centrality for all the nodes of the network, we see both examples of underestimation and overestimation when comparing fixed conduction velocity with the estimated velocity distribution.

**Fig. 4:**
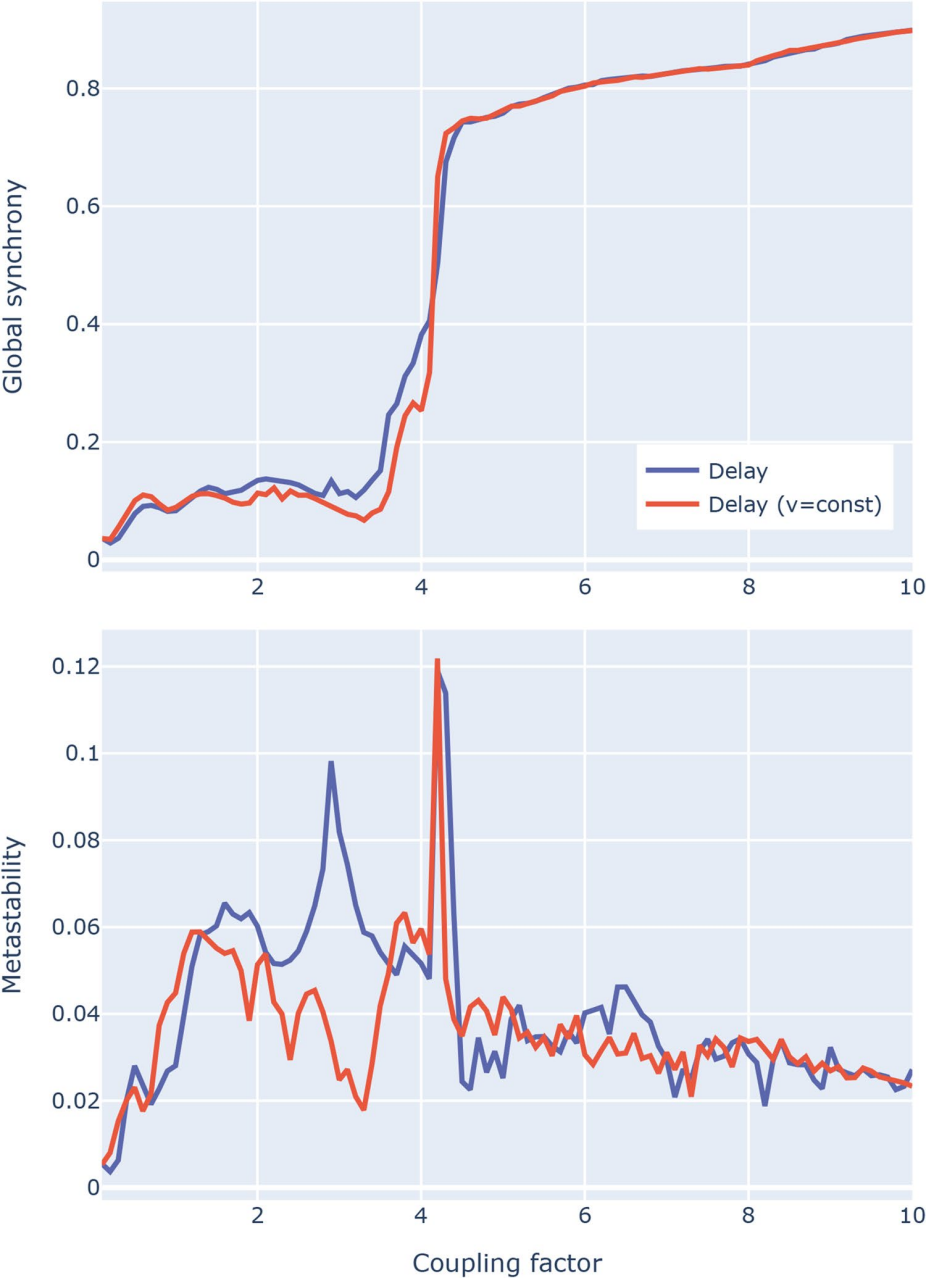
Comparisons between conduction delay distribution with the constant velocity approximation using simulations based on the Kuramoto model: in both cases, the resulting global synchrony (top) and the metastability (bottom) show similar trends over the coupling factor range

The final question we wanted to answer is about a more general dynamical behavior using the delay distributions: do these edge- and node-specific differences lead to a substantial difference when we simulate oscillation phenomena? To answer this question, we used the Kuramoto model (Kuramoto, 1984). In this model, each brain region is represented as an oscillator and characterized mainly by its phase (Breakspear et al., 2010). Each oscillator adjusts its own phase according to the phases of the other oscillators through the existing connections between them. Although the original formulation of the Kuramoto model does not assume delays in the interactions between oscillators, subsequent iterations explored the effect of such delays (Rodrigues et al., 2016; Yeung & Strogatz, 1999). Leveraging this delay formulation, we computed both global synchrony and metastability measures for a range of coupling factors (Fig. 4). We observed negligible differences between models based on either delay estimation, with both distributions leading to very similar synchronization patterns: the global synchrony measures show the critical regime (i.e., where the oscillators become rapidly synchronized) for the same coupling factor values, and likewise the metastability measures reach their peak in the same range.

## Discussion

In this work, we leveraged microstructural measures estimated from MRI to study the conduction delay distribution in the whole-brain structural network. Our approach focused on conduction velocity and its main two determinants: the axonal diameter and the g-ratio (Drakesmith et al., 2019). The related MRI-based measures have received a lot of attention over the years, and several studies focused on their validation using histology as ground truth (Mancini et al., 2020; Veraart et al., 2020), and on their potential use as biomarkers in pathology (Yu et al., 2019). Previous studies have started studying the relationship between conduction properties and MRI-based measures in two specific pathways, the corpus callosum (S. Berman et al., 2019; Horowitz et al., 2015) and the optic radiation (Shai Berman et al., 2020; Takemura et al., 2020; You et al., 2019). To the best of our knowledge, this is the first attempt to combine them to estimate conduction velocity and delay distributions across the whole brain.

To understand the conduction delay distribution in terms of its determinants, we first described how each microstructural measure and the resulting conduction velocity change as a function of connection length. We observed that both the axonal diameter and the conduction velocity follow an overall constant trend, and therefore, the computed conduction delays showed a remarkably linear relationship with the connection length. Not only does this result support the approximation of constant conduction velocity used in several computational studies (Cabral et al., 2011, 2014), but it is also in line with the few studies that quantified delays through invasive recordings, in macaques (Shimono & Hatano, 2018) and in humans (Silverstein et al., 2020). Specifically, Shimono and Hatano relied on existing electrocorticography (ECoG) datasets to estimate cortico-cortical delays (Shimono & Hatano, 2018), while Silverstein and colleagues used direct electrical stimulation in concurrency with recording intracranial electroencephalography (iEEG) (Silverstein et al., 2020). Given this qualitative agreement, it becomes clear that for quantitative predictions the next step for computational studies is defining a rigorous method to set either the proportionality constant used to estimate conduction velocity or, equivalently, the approximate constant velocity.

It is important to place the microstructural MRI measures in the appropriate context. Despite the use of an ultra-high gradient MRI scanner, we still have to deal with the diffusion resolution limit dictated by the current hardware (Huang et al., 2015; Jones et al., 2018). Specifically, with gradients of 300 mT/m, we are able to measure axons with a diameter of 2–3 μm (Drobnjak et al., 2016; Nilsson et al., 2017). In line with this limit, further MRI simulations for two specific microstructural approaches, AxCaliber (Drakesmith et al., 2019) and the spherical mean model (Fan et al., 2020), showed larger estimation errors for smaller axons. These results prevent current approaches to fully characterize the axonal diameter distribution in the central nervous system, but ultra-high gradient MRI still allows us to study the upper tail of such distribution. Histological validation work from Veraart and colleagues (Veraart et al., 2020) showed that we are sensitive to the fourth order moment of the axonal diameter distribution, and therefore we are only taking into account the largest axons. It follows that in these distributions most axons have a much smaller diameter and very few axons have the large ones we are sensitive at ultra-high gradients. Nevertheless, such large axons result in high conduction velocity, and therefore play an important role the propagation of faster oscillations (Ivanov et al., 2019). For a complete characterization of conduction phenomena in the brain, we would instead need a conduction velocity distribution for each connection. A similar limitation holds for the relative myelin content, the g-ratio: the so-called “aggregate” (i.e. voxel-level) g-ratio is approximately axon-area weighted (Campbell et al., 2018; West et al., 2016), and therefore larger axons have a greater weight.

There is also another interesting consideration for the role of myelin to make: the g-ratio, as estimated from MRI, does not seem to visibly alter the overall conduction velocity, whose distribution instead closely resembles that of the axonal diameter—at least for healthy subjects. This is expected: Drakesmith and colleagues (Drakesmith et al., 2019) already showed through simulations that the g-ratio, and therefore the myelin content, seems to have a limited role in modulating conduction velocity. While combining the axonal diameter and the g-ratio accounts for the 85.1% of the variance in conduction velocity, the g-ratio variance contribution is much smaller (around 12%). When using both the Waxman and the Rushton models, the largest myelin-driven effects on conduction velocity are predicted for very large axons (around 7–10 μm) and for very low g-ratio values (around 0.4–0.6). However, it is important to stress that this limited role may be the result of how we currently model conduction phenomena—in most cases through single-neuron models (Arancibia-Cárcamo et al., 2017). As some studies have started to show (H. Schmidt et al., 2021; Sheheitli & Jirsa, 2020), the presence of multiple axons running in parallel and the potential interactions among them may be an important factor to take into account. Another limitation is that the Rushton and Waxman models (Rushton, 1951; Waxman & Bennett, 1972) have been derived from peripheral nerve experiments and may have reached their limits when it comes to myelinated axons in the brain.

To further explore the validity of the conduction velocity approximation, we compared the conduction delay distribution computed from the axonal diameters with the one obtained with a constant conduction velocity estimated through linear regression. From a network modeling perspective, there are some differences for the overall delays between regions—as assessed with shortest path lengths—and for each region’s role in fast connections—as assessed with betweenness centrality. Despite these differences, simulating the actual dynamics with the Kuramoto model shows remarkably similar results in both cases. We conclude that, while the constant velocity approximation can be considered valid for whole-brain simulations, the observed differences may play a role when focusing the attention on specific regions or pathways.

As we relied on the concept of path for graph-based measures, it is necessary to discuss the methodological choice of how to define paths in brain networks. Given two nodes in a network, there are several approaches to characterize how the network topology constrains any communication process. Shortest paths have been widely used to characterize the brain structure and function (Fornito et al., 2016), however this approach assumes that each node has a global knowledge of the network topology—and so far there is little evidence that supports this assumption. Alternative approaches are diffusion processes and navigation, relying on the assumptions that communication processes are based, respectively on dispersive information flows (Mišić et al., 2015) and on greedy routing strategies (Seguin et al., 2018). In this study, we decided to rely on shortest paths as our goal was to approximate the shortest delay between two given regions for both the cases of estimated delay distribution and fixed conduction velocity.

Despite the overall agreement with primate electrophysiological studies, there is a discrepancy with rodent studies reporting constant delays in subcortico-cortical connections (Pelletier & Paré, 2002; Salami et al., 2003). In a study on thalamo-cortical connections (Salami et al., 2003), Salami and colleagues showed that stimulating the ventrobasal nucleus of the thalamus in ex vivo mice brain tissue evoked cortical responses with the same delays as stimulating the white matter between the thalamus and the cortex. Intriguingly, this feature was not present in mice with a lower postnatal age. These results deserve further investigations, as it is not clear if this feature may be limited to specific bundles of myelinated axons (Kimura & Itami, 2009). As explained by the authors, as a result of development the ventrobasal-white matter connection becomes ten times faster and therefore becomes negligible compared the ventrobasal-cortical connection. However, this does not tell us if distinct thalamo-cortical connections, with different lengths, would present the same overall delay, as later suggested (Pajevic et al., 2014). Extensive work on macaque brains from Innocenti and colleagues (Innocenti et al., 2013; Tomasi et al., 2012) did not report any correlation between diameter and length. Other studies reported isochronic properties in rodents for the amygdala (Pelletier & Paré, 2002) and the prefrontal cortex (McDougall et al., 2018), but again focusing on specific bundles. In any case, at the whole-brain level our results point out that constant delays seem unlikely to generally occur. As we have already discussed in detail, we are measuring the largest axons in each bundle and the resulting delays scale linearly with the connection length. This result implies that the rest of the axons in each bundle—smaller and, therefore, slower—would not be able to compensate for different connection lengths.

One final limitation we need to disclose is the limited number of subjects. As these measures required a dedicated ultra-high gradient MRI scanner and a substantial amount of scanning time, it is not possible to use any of the current publicly available large datasets to estimate the same measures. Nevertheless, to further support our conclusions, we took into account also the trends observed at the single-subject level, which replicate the results obtained at the group level.

## Conclusions

To summarize, using microstructural measures estimated from MRI we were able to estimate the conduction velocity and delay distributions of white matter connections across the whole brain. These results support in general the current approximation of constant conduction velocity for simulation purposes, but also suggests that velocity distribution may contribute to provide a more detailed picture for specific areas and pathways. Overall, leveraging microstructural measures has the potential to further refine network models of the brain.

## Methods

### Data acquisition and general pre-processing

Fourteen healthy subjects (6/8 M/F, mean age/SD: 40.92/13.88) were scanned using a dedicated ultra-high gradient MRI scanner (Magnetom Connectom; Siemens, Erlangen, Germany), with a maximum gradient strength of 300 mT/m and a maximum slew rate of 200 T/m/s. The protocol included (Yu et al., 2019):

- T1-weighted multi-echo acquisition (MPRAGE; 1-mm isotropic resolution; TEs = 1.15, 3.03, 4.89, 6.75 ms; TR = 2530 ms; TI = 1100 ms; parallel imaging acceleration factor R = 3, flip angle: 7°);
- Multi-shell diffusion-weighted acquisition (spin-echo single-shot EPI sequence; 2-mm isotropic resolution; TE = 77 ms; TR = 3600 ms; R = 2; anterior-to-posterior encoding; diffusion gradient pulse duration: 8 ms; diffusion times: 19 ms, 49 ms; 16 b-values, applied in 32 directions for b-values < 2300 s/mm^2^ and 64 directions for b-values > 2300 s/mm^2^; maximum b-value 17,800 s/mm^2^; 5 b = 0 volumes acquired with reversed-phase encoding direction);
- MTV acquisition (multiple flip angle spoiled gradient-echo 3D-FLASH sequence; 1-mm isotropic resolution; TE = 2.74; TR = 20 ms; flip angles: 4°, 10°, 20°);
- B1 mapping, performed using actual flip angle imaging, which was performed with a dual TR steady-state gradient-echo sequence (flip angle 53 degrees, TR = 11 and 33 ms, TE = 4.93, FOV 192 × 192 × 168 mm, matrix size = 64 × 64 × 56).

The study was approved by the Mass General Brigham institutional review board and is compliant with the Health Insurance Portability and Accountability Act guidelines. All participants provided written informed consent.

The pre-processing of the data, already described in a previous study (Yu et al., 2019), included: correcting diffusion data for gradient nonlinearity; processing diffusion data with the FSL tools *topup* and *eddy* to correct for, respectively, susceptibility- and eddy current-induce distortions; correcting MTV data for B1 inhomogeneity; rigid alignment of MTV and diffusion data to the T1-weighted volumes.

### Microstructural measures estimation

To estimate axonal diameter and the related tissue compartments, we relied on the spherical mean model (Fan et al., 2020; Kaden et al., 2016). Briefly, a three-compartment model was incorporated into the spherical mean framework to estimate: the axonal diameter; the relaxation-weighted restricted (*f*_*r*_) and hindered (*f*_*h*_) volume fractions; and the free water diffusion (*f*_*csf*_) volume fraction. More details are provided in a previous study (Fan et al., 2020).

The myelin content was estimated from the MTV data (Mezer et al., 2013) and calculated as:

$$MTV= 1- \frac{PD}{{PD}_{f}}$$

where *PD*_*f*_ represents the proton density of free water. We then combined myelin and diffusion-based measures to compute the g-ratio. Assuming that MTV approximates MVF (S. Berman et al., 2018; Duval et al., 2017), we computed the axonal volume fraction as:

$$AVF=\left(1-MTV\right)\times (1- {f}_{csf})\times {f}_{r}$$

The g-ratio was then calculated as proposed by Stikov and colleagues (Stikov et al., 2015):

$$g= \sqrt{\frac{1}{1+\frac{MTV}{AVF}}}$$

Once all the volumes were in the diffusion space, we computed a voxel-wise measure of conduction velocity using the formula proposed by Rushton (Rushton, 1951):

$$v=k\bullet d\bullet \sqrt{-\mathrm{ln}g}$$

where *k* is a proportionality constant. We chose this model as it has shown the highest agreement with single-neuron simulations (Drakesmith et al., 2019). On the basis of those simulations, we set *k* to 7⋅10^6^ s^−1^.

### Structural connectivity

The T1-weighted data were processed using FreeSurfer (version 6.0) for tissue classification (Dale et al., 1999) and parcellation with the Desikan-Killiany atlas (Desikan et al., 2006) (85 ROIs, including cortical and subcortical areas, the cerebellum and the brainstem). As an additional parcellation scheme, the Lausanne atlas (Cammoun et al., 2012) was used (scale 250, with 463 ROIs), reconstructed from the FreeSurfer output by means of the *EasyLausanne* tool (https://github.com/mattcieslak/easy_lausanne—commit hash 86892c9), a stripped-down version of the Connectome Mapper (Daducci et al., 2012).

For structural connectivity purposes, we used the subset of the diffusion data acquired with a diffusion time of 49 ms and with the following b-values: 0, 2300, 4250. To align the anatomical parcellation to the diffusion data, the boundary-based registration (BBR) (Greve & Fischl, 2009), as implemented in FSL FLIRT (version 6.0), was used to estimate a rigid transformation from the diffusion space to the anatomical space. Using the inverse of this transformation, the anatomical data was aligned with the diffusion data. For subsequent fiber orientation reconstruction and streamline tracking, multi-tissue multi-shell constrained spherical deconvolution (Jeurissen et al., 2014) and anatomical-constrained tractography (Smith et al., 2012) as implemented in MRtrix3 (version 3.0.0) (Tournier et al., 2019) were used, with the following parameters: probabilistic algorithm (iFOD2); backtracking; cropping at the interface between white matter and gray matter; dynamical seeding; minimum streamline length of 10 mm; maximum streamline length of 250 mm; angular threshold of 30 degrees; target number of streamlines of 1 million. The microstructural measures of interest (axonal diameter, g-ratio, MTV, conduction velocity) were then mapped to the resulting tractogram using the *tcksample* tool from MRtrix3.

Finally, for each subject we reconstructed six connectivity matrices, respectively, weighted using: the number of streamlines (NOS) interconnecting each pair of regions; the average length of the streamline subset from each pair; for all the microstructural measures, the average computed across each bundle, where a bundle is the set of streamlines interconnecting a pair of regions. For subsequent analyses, we excluded cerebellum and brainstem ROIs. From the individual NOS-weighted connectivity matrices, we estimated a group matrix averaging all the connections with more than 4 streamlines in at least 9 subjects (~ 60% subjects) to find a balance between reducing false positives and limiting false negatives (de Reus & van den Heuvel, 2013).

In the fundamental characterization of the relationships between different measures, we used linear regression as implemented in the Python package *scikit-learn* (version 0.22.2.post1) (Pedregosa et al., 2011) and Pearson correlation as implemented in the Python package *numpy* (version 1.18.4) (Harris et al., 2020). All the figures were generated using *plotly* (version 4.14.3).

### Graph measures and Kuramoto simulations

Conduction delays provide a meaningful way to define how to weight a network in terms of white matter conduction properties. To understand how these delays influence integration across the whole brain, we relied on the concept of shortest path from graph theory: the shortest path in a network is the sequence of edges between a given pair of nodes with the shorter distance associated – in this specific case, the distance is defined relying on the delay as a weight. For each pair of regions, we computed the shortest paths using the Dijkstra’s algorithm (Dijkstra, 1959) as implemented in the Python package *bctpy* (version 0.5.2), which is based on the Brain Connectivity Toolbox (Rubinov & Sporns, 2010). To also clarify the specific role of each region in these paths, we computed the betweenness centrality (Freeman, 1977), which represents the proportion of shortest paths passing through a given node and can be calculated as:

$${C}_{B}\left(i\right)= \frac{1}{(N-1)(N-2)}\sum_{h\ne i,h\ne j,j\ne i}\frac{{\rho }_{hj}(i)}{{\rho }_{hj}}$$

where *i* is the target node, *j* and *h* are any other node in the network, *ρ*_*hj*_*(i)* is the number of shortest paths between h and j passing through *i* and *ρ*_*hj*_ is the total number of shortest paths between *h* and *j*.

To finally gain an overall picture of the dynamical behavior resulting from the observed delay distributions, we used simulations based on the Kuramoto model (Kuramoto, 1984) with the introduction of delays (Yeung & Strogatz, 1999).

In this model, each region is represented as an oscillator and is coupled to the others on the basis of the connectivity matrix. The dynamical behavior can be described through the following differential equation:

$$\frac{d{\theta }_{n}}{dt}=\omega +k\sum_{p=1}^{N}{C}_{np}\mathrm{sin}({\theta }_{p}\left(t-{\tau }_{np}\right)-{\theta }_{n}(t)),\; n=1, \dots , N$$

where *θ*_*n*_ is phase of the *n*-th oscillator, *ω* is the natural frequency, *k* is the coupling factor, *C*_*np*_ represents the presence (1) or absence (0) of coupling between the oscillators *n* and *p* accordingly to the connectivity matrix, and *τ*_*np*_ is the delay between *n* and *p*. As one can observe, the natural frequency is the same among the oscillators, while the phase characterizes each oscillator and couples the interconnected ones.

To assess the synchronization, we represent the phases as a complex variable – *z(t)* – and we then use the order parameter *r(t)*, which is the modulus of *z(t)*:

$$z\left(t\right)= \frac{1}{N}\sum_{j=1}^{N}{e}^{i{\theta }_{j}(t)}=r(t){e}^{i\Phi (t)}$$

where *r(t)* measures phase uniformity and *Φ(t)* describes the phase of the global ensemble (Cabral et al., 2014). This representation makes easier to assess global synchronization: from the previous formula it follows that *r(t)* can vary from 0 (for a heterogeneous system where the oscillators behave incoherently) to 1 (for a fully synchronized system where the oscillators have the same phase). We can then derive from *r(t)* two specific measures to describe the synchronization of the system of oscillators: the average (over time) of *r(t)*, which represents the global synchrony; and the standard deviation of *r(t)*, which measures how much the overall phase coupling fluctuates over time and therefore describes the phase configuration stability – the so-called metastability (Deco et al., 2017). For a more comprehensive review on the Kuramoto model the interested reader is referred to the paper by Rodrigues and colleagues (Rodrigues et al., 2016).

To run the simulations, we used a modified version of the Network Model Toolbox (original code available here: https://github.com/juanitacabral/NetworkModel_Toolbox; modified version available here: https://github.com/matteomancini/NetworkModel_Toolbox; commit hash 0bccd1e) to easily set the parameters of interest (delay distribution, time step, simulation length, random number generator seed). We set the natural frequency to 40 Hz and the coupling factor in the range between 0.1 and 10 (both extremes included) with a step of 0.1: for each coupling factor we ran 100 simulations, each simulation going from 1 ms to 1 s with a time step of 1 ms. To compute the synchronization parameters, we discarded the initial transient samples and used the range between 300 and 700 ms (de Lange et al., 2019; R. Schmidt et al., 2015).

### Single-subject analysis

One of our goals was to check if the patterns observed at the group level were also present at the subject level. The main challenge of assessing single-subject structural connectivity is related to presence of false positives (Maier-Hein et al., 2017). To tackle this issue, we used COMMIT2 as an additional step in our processing pipeline (Schiavi et al., 2020). COMMIT2 (where COMMIT stands for ‘convex optimization modeling for microstructure informed tractography’) leverages anatomical priors to filter out spurious streamlines that do not reflect the brain fiber organization. Briefly, using the Python package *dmri-commit* (version 1.4.5), we first ran COMMIT to fit a stick-ball model to the data and to estimate the streamline weights without introducing any prior. We then subdivided the tractogram in bundles, and for each bundle we calculated its individual weight (Schiavi et al., 2020). We finally fitted COMMIT2 using the regularization term obtained combining the subdivision in bundles, each bundle’s individual weight and the regularization parameter *λ* (set to 5⋅10^–4^). The resulting filtered tractograms were then used as already described to compute the connectivity matrices – with the only additional step of filtering out any bundle with an average diameter smaller than 1 μm.

## Supplementary Information

Below is the link to the electronic supplementary material.

Supplementary file1 (PDF 15959 KB)

## Data Availability

The dataset used in this study is publicly available at the following link: https://doi.org/10.6084/m9.figshare.15141909.

## Code availability

The code to reproduce the analysis performed in this study is publicly available at the following link: https://github.com/matteomancini/dissecting-conduction-delays.
