# Supplementary material for: Dissecting whole-brain conduction delays through MRI microstructural measures
Source: Brain Struct Funct. 2021 Aug 14;226(8):2651–63. doi: 10.1007/s00429-021-02358-w (PMC8448685; doi:10.1007/s00429-021-02358-w)
Supplement: Supplementary file 1 — Supplementary file1 (PDF 15959 KB) [file 429_2021_2358_MOESM1_ESM.pdf]

Dissecting whole-brain conduction delays through MRI microstructural measures

Matteo Mancini, Qiyuan Tian, Qiuyun Fan, Mara Cercignani, Susie Y. Huang

Supplementary material

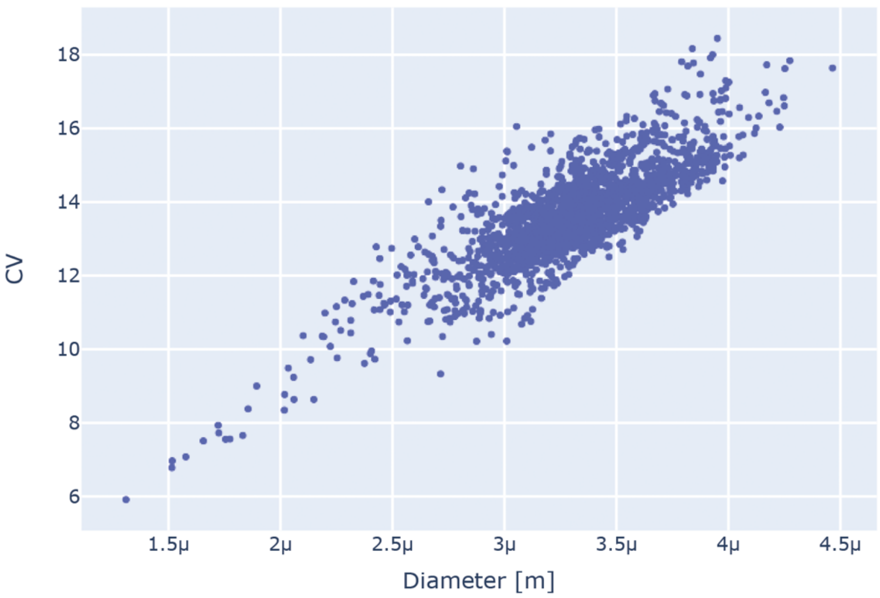

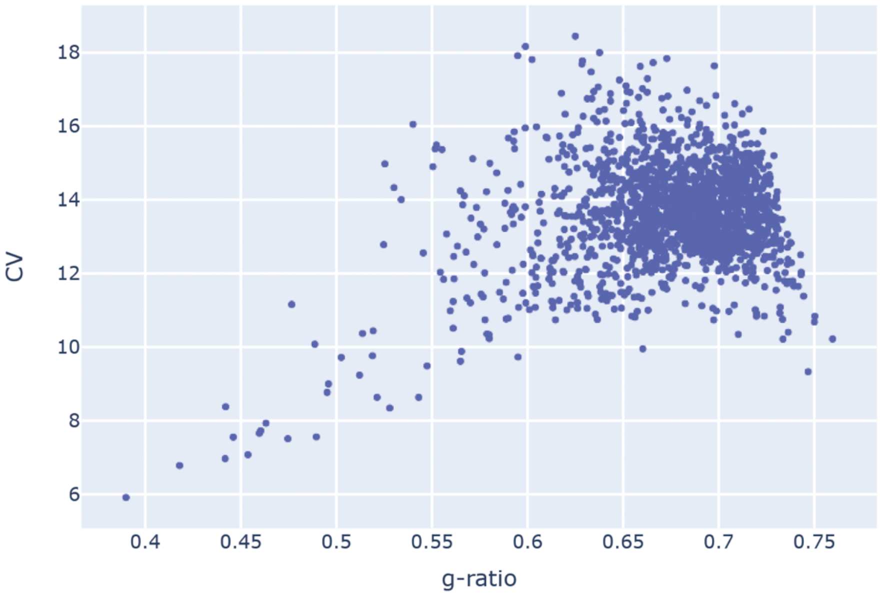

Figure S1 Relationships between the conduction velocity and its main determinants: the axonal diameter (top) and the g-ratio (bottom). The diameter-CV relationship has an evident linear trend, while the g-ratio-CV one shares some similarities with the g-ratio-diameter trend (Figure 2).

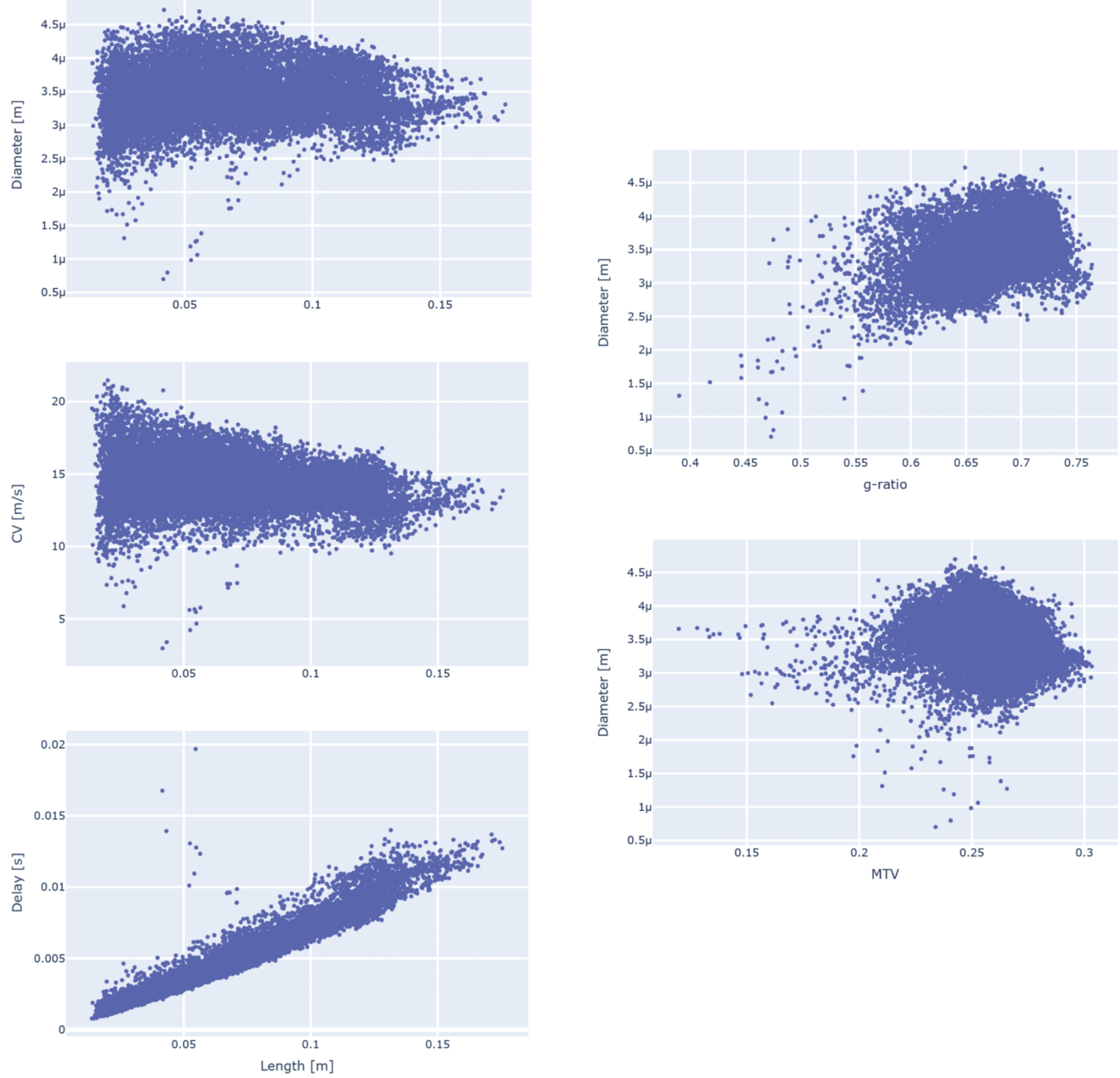

Figure S2 Relationships between microstructural measures using the Lausanne atlas: on the left distributions of the axonal diameter (top), conduction velocity (middle) and delay (bottom) as a function of the connection length; on the right, axonal diameter distributions as a function of the g-ratio (top) and MTV (bottom Each point in the scatterplots represent a connection in the group network.

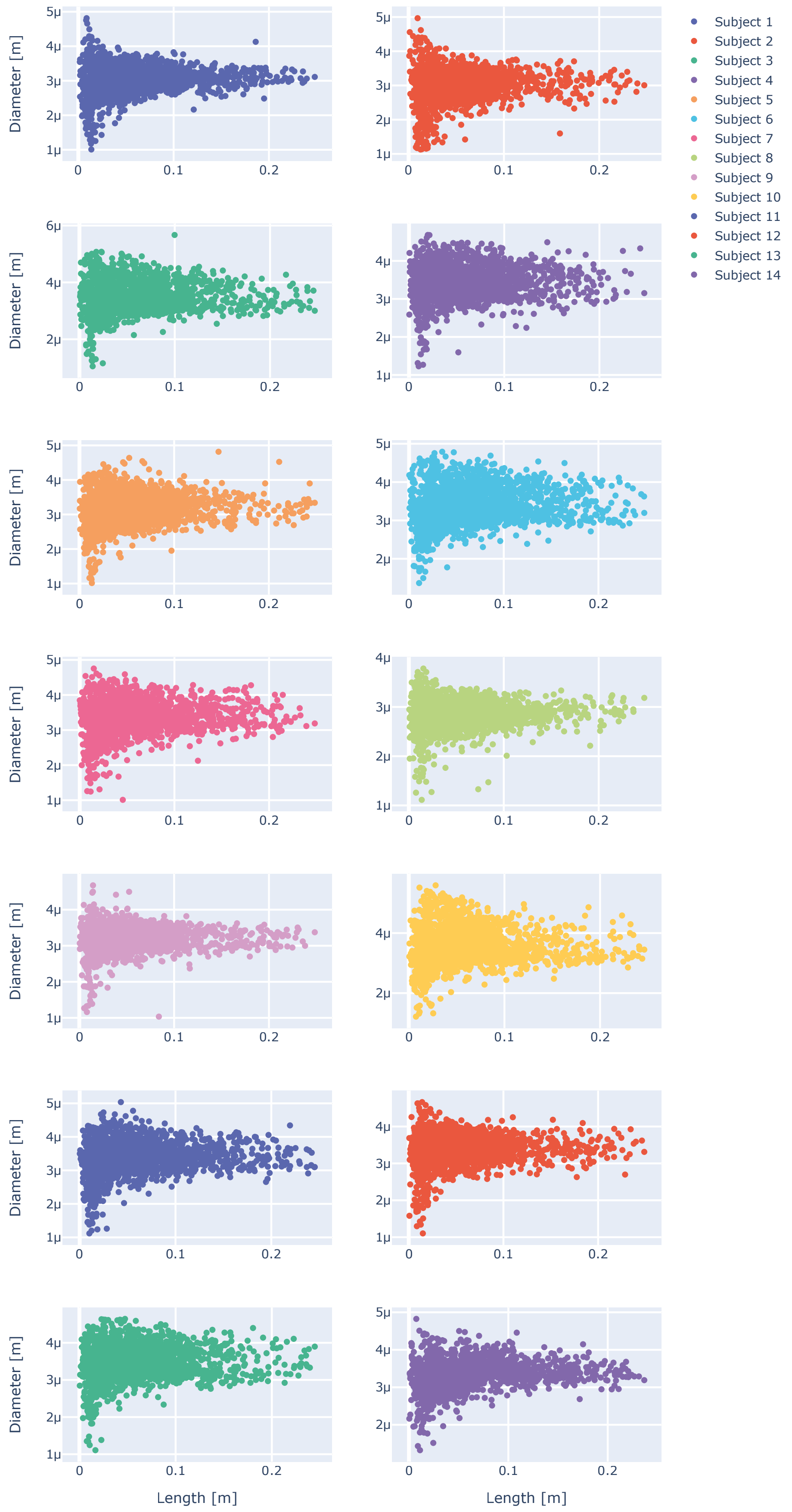

Figure S3 Single-subject distribution of the axonal diameter distribution as a function of length.

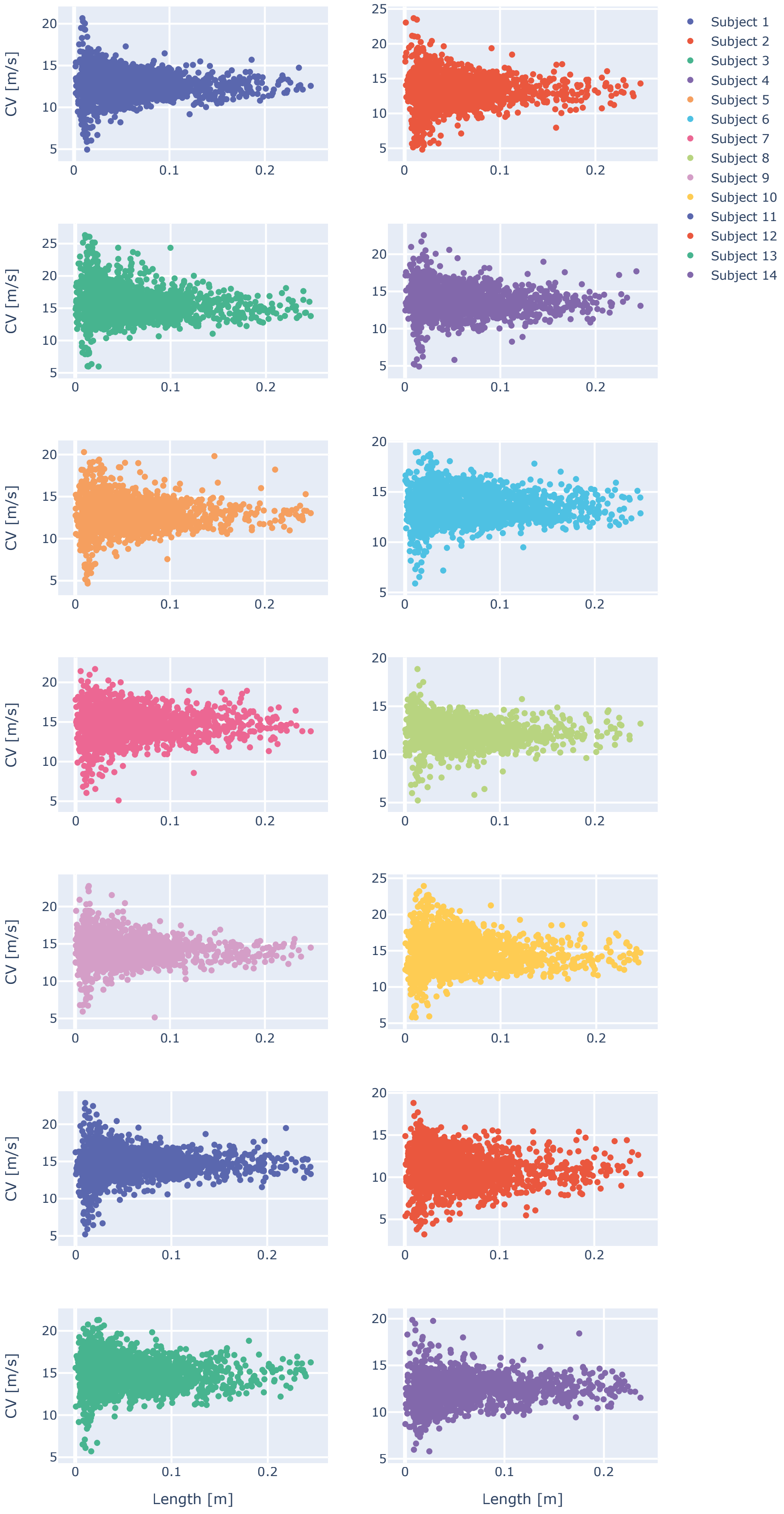

Figure S4 Single-subject distribution of the conduction velocity distribution as a function of length.

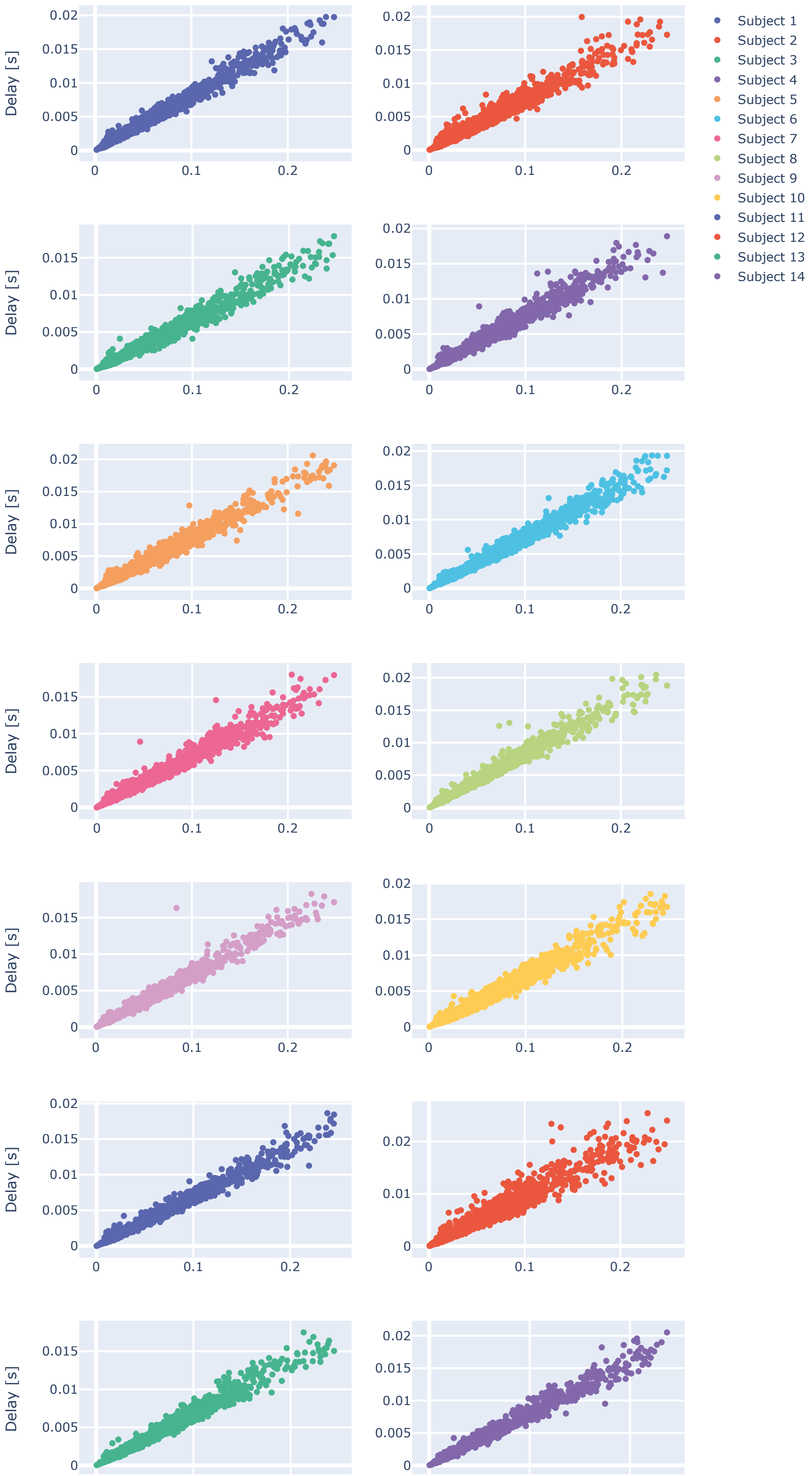

Figure S5 Single-subject distribution of the conduction delay distribution as a function of length.

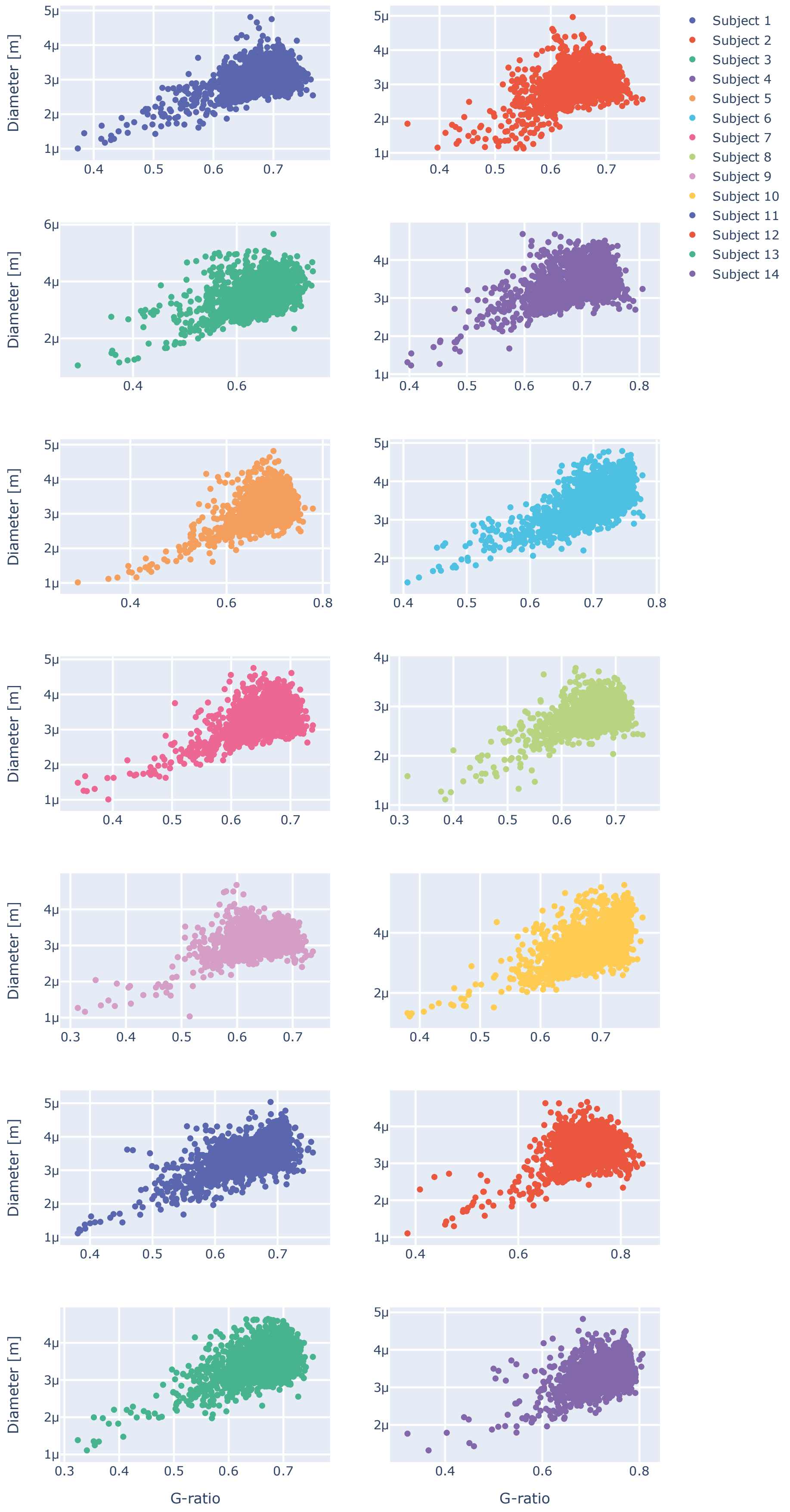

Figure S6 Single-subject distribution of the axonal diameter distribution as a function of the g-ratio.

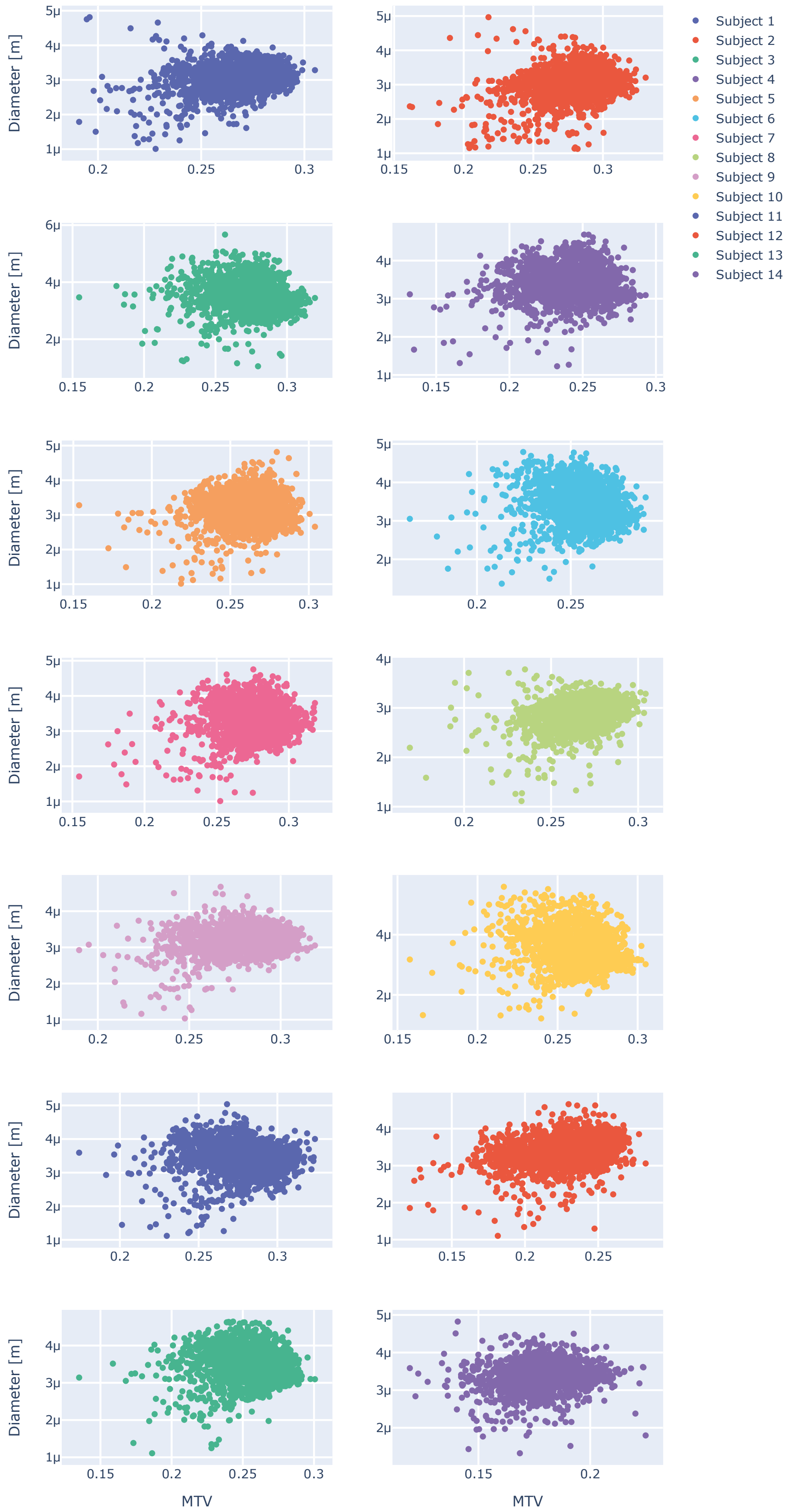

Figure S7 Single-subject distribution of the axonal diameter distribution as a function of MTV.

Table S1 List of FreeSurfer ROIs from the Desikan-Killiany atlas in the same order as in fig. 3.

Cortical/Subcortical	Hemisphere	FreeSurfer label
Cortical	Left	bankssts
Cortical	Left	caudalanteriorcingulate
Cortical	Left	caudalmiddlefrontal
Cortical	Left	cuneus
Cortical	Left	entorhinal
Cortical	Left	fusiform
Cortical	Left	inferiorparietal
Cortical	Left	inferiortemporal
Cortical	Left	isthmuscingulate
Cortical	Left	lateraloccipital
Cortical	Left	lateralorbitofrontal
Cortical	Left	lingual
Cortical	Left	medialorbitofrontal
Cortical	Left	middletemporal
Cortical	Left	parahippocampal
Cortical	Left	paracentral
Cortical	Left	parsopercularis
Cortical	Left	parsorbitalis
Cortical	Left	parstriangularis
Cortical	Left	pericalcarine
Cortical	Left	postcentral
Cortical	Left	posteriorcingulate
Cortical	Left	precentral
Cortical	Left	precuneus
Cortical	Left	rostralanteriorcingulate
Cortical	Left	rostralmiddlefrontal
Cortical	Left	superiorfrontal
Cortical	Left	superiorparietal
Cortical	Left	superiortemporal
Cortical	Left	supramarginal
Cortical	Left	frontalpole
Cortical	Left	temporalpole
Cortical	Left	transversetemporal
Cortical	Left	insula
Subcortical	Left	Thalamus-Proper
Subcortical	Left	Caudate
Subcortical	Left	Putamen
Subcortical	Left	Pallidum
Subcortical	Left	Hippocampus
Subcortical	Left	Amygdala
Subcortical	Left	Accumbens-area
Subcortical	Right	Thalamus-Proper
Subcortical	Right	Caudate

Subcortical	Right	Putamen
Subcortical	Right	Pallidum
Subcortical	Right	Hippocampus
Subcortical	Right	Amygdala
Subcortical	Right	Accumbens-area
Cortical	Right	bankssts
Cortical	Right	caudalanteriorcingulate
Cortical	Right	caudalmiddlefrontal
Cortical	Right	cuneus
Cortical	Right	entoRightinal
Cortical	Right	fusiform
Cortical	Right	inferiorparietal
Cortical	Right	inferiortemporal
Cortical	Right	isthmuscingulate
Cortical	Right	lateraloccipital
Cortical	Right	lateralorbitofrontal
Cortical	Right	lingual
Cortical	Right	medialorbitofrontal
Cortical	Right	middletemporal
Cortical	Right	parahippocampal
Cortical	Right	paracentral
Cortical	Right	parsopercularis
Cortical	Right	parsorbitalis
Cortical	Right	parstriangularis
Cortical	Right	pericalcarine
Cortical	Right	postcentral
Cortical	Right	posteriorcingulate
Cortical	Right	precentral
Cortical	Right	precuneus
Cortical	Right	rostralanteriorcingulate
Cortical	Right	rostralmiddlefrontal
Cortical	Right	superiorfrontal
Cortical	Right	superiorparietal
Cortical	Right	superiortemporal
Cortical	Right	supramarginal
Cortical	Right	frontalpole
Cortical	Right	temporalpole
Cortical	Right	transversetemporal
Cortical	Right	insula
